# The Cost-Utility of CT Angiography and Conventional Angiography for People Presenting with Intracerebral Hemorrhage

**DOI:** 10.1371/journal.pone.0096496

**Published:** 2014-05-13

**Authors:** Richard I. Aviv, Adam G. Kelly, Babak S. Jahromi, Curtis G. Benesch, Kate C. Young

**Affiliations:** 1 Department of medical Imaging, University of Toronto, Division of Neuroradiology Sunnybrook Health Sciences Centre, Toronto, Ontario, Canada; 2 Department of Neurology, University of Rochester, Rochester, New York, United States of America; 3 Department of Neurosurgery, University of Rochester, Rochester, New York, United States of America; 4 Department of Imaging Sciences, University of Rochester, Rochester, New York, United States of America; Johns Hopkins Hospital, United States of America

## Abstract

**Objective:**

To determine the optimal imaging strategy for ICH incorporating CTA or DSA with and without a NCCT risk stratification algorithm.

**Methods:**

A Markov model included costs, outcomes, prevalence of a vascular lesion, and the sensitivity and specificity of a risk stratification algorithm from the literature. The four imaging strategies were: (a) CTA screening of the entire cohort; (b) CTA only in those where NCCT suggested a high or indeterminate likelihood of a lesion; (c) DSA screening of the entire cohort and (d) DSA only for those with a high or indeterminate suspicion of a lesion following NCCT. Branch d was the comparator.

**Results:**

Age of the cohort and the probability of an underlying lesion influenced the choice of optimal imaging strategy. With a low suspicion for a lesion (<12%), branch (a) was the optimal strategy for a willingness-to-pay of $100,000/QALY. Branch (a) remained the optimal strategy in younger people (<35 years) with a risk below 15%. If the probability of a lesion was >15%, branch (b) became preferred strategy. The probabilistic sensitivity analysis showed that branch (b) was the optimal choice 70–72% of the time over varying willingness-to-pay values.

**Conclusions:**

CTA has a clear role in the evaluation of people presenting with ICH, though the choice of CTA everyone or CTA using risk stratification depends on age and likelihood of finding a lesion.

## Introduction

Following the diagnosis of intracranial hemorrhage (ICH) on non contrast CT (NCCT), the American Heart Association guidelines recommend the use of non-invasive vascular imaging such as CT angiography (CTA) to evaluate for the presence of underlying vascular lesions.[1] The guidelines, however, limit this recommendation to ICH where a clinical or radiological (based on non-contrast CT - NCCT) suspicion of an underlying lesion exists.

Traditionally, the role of NCCT has been confined to ICH diagnosis and facilitation of management decisions pertaining to ICH-related complications. Additionally, radiological and clinical features have been described that suggest the possibility of an underlying structural lesion.[2]–[5] Only one prospective study has quantified NCCT performance for the detection of an underlying secondary vascular lesion and demonstrated a modest sensitivity and specificity of 77% and 84% respectively. A recent retrospective study, stratified NCCT scan appearances into low, indeterminate and high risk and reported the performance of NCCT for predicting an underlying vascular lesions. Acknowledging that both indeterminate and high risk NCCT scans require further vascular workup, the study demonstrated a sensitivity and specificity of 96% and 33% respectively.[2]

Pure reliance on clinical and NCCT features to determine the risk of an underlying lesion is an historic approach stemming from prior practice where only digital subtraction angiography (DSA) was available to confirm clinical or NCCT suspicion.[6] Fear of DSA- associated morbidity and mortality led to the procedure being performed only in a limited and highly selected population where the procedural risks were outweighed by the perceived benefit of the confirmatory diagnosis. These fears, however, likely contributed to a selection and verification bias that has distorted the true prevalence of secondary vascular lesions. Although safety and accuracy of CTA is well established for aneurysm, its performance in non-subarachnoid ICH is only more recently established. There are now numerous publications demonstrating sensitivities and specificities of CTA for secondary vascular causes ranging from 92–100%.[2]–[5], [7] We have previously proposed that CTA screening should be implemented in all ICH patients irrespective of NCCT risk stratification or perceived clinical risk.[6] However, acknowledging the widespread reliance on NCCT to risk stratify ICH presentations, we sought to determine the optimal angiographic strategy for ICH work-up by exploring the future costs and outcomes related to CTA and DSA use without and with a NCCT risk stratification algorithm.

## Methods

### Model Description

**Figure 1:**
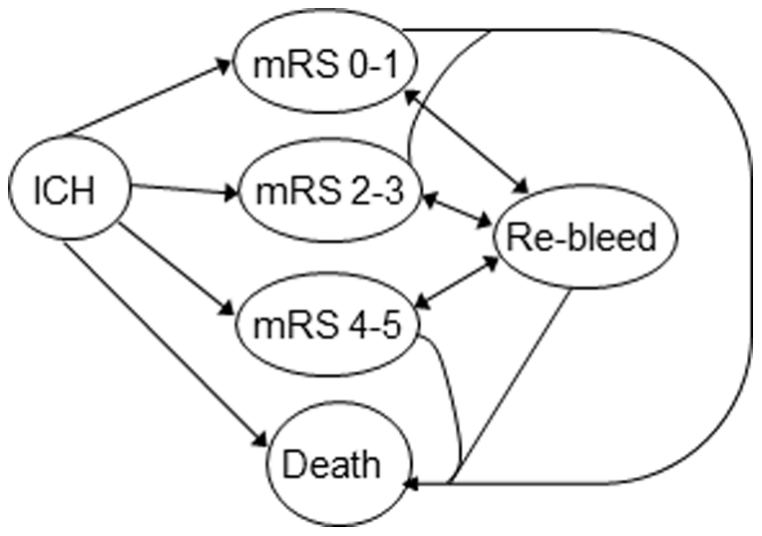
Influence Diagram. The health states following presentation with primary ICH or secondary ICH are depicted.

We developed a Markov model to predict the future costs and health-related outcomes of different imaging strategies (Figure 1). Markov modeling allowed for future estimation of outcomes based on several potential health states after the initial event. The Markov cycle length was one year. The time horizon was the lifetime of the cohort.

There were four branches at the decision node: (a) immediate CTA screening of the entire cohort; (b) A stratified CTA approach - CTA performed only in those where the NCCT suggested a high or indeterminate (medium) likelihood of an underlying lesion. Patients with a low suspicion of an underlying lesion on NCCT do not undergo CTA vascular imaging [8]; (c) DSA screening of the entire cohort and (d) A stratified DSA approach - DSA performed where the NCCT suggested a high or indeterminate likelihood of an underlying lesion. As above, patients with a low suspicion of an underlying lesion on NCCT would not undergo DSA vascular imaging. Branch (d) was the comparator or the strategy against which the other three were compared. The sensitivity and specificity of CTA and NCCT to identify a lesion were derived from prior studies (Table 1).[2], [3], [5], [7], [8].

**Table 1:** Model inputs.

Parameter	Base Case	Range	Distribution for PSA	References
Initial Age	40	10–80	Normal (SD = 12.5)	Author estimate
Sensitivity of CTA	0.96	0.8–1.0	Beta	[2], [3], [5], [7]
Specificity of CTA	0.99	0.8–1.0	Beta	[2], [3], [5], [7]
Sensitivity of NCCT high/indeterminate risk	0.95	0.7–1.0	Beta	[45]
Specificity of NCCT high/indeterminate risk	0.35	0–0.6	Beta	[45]
**Probabilities, %**				
mRS after Primary ICH			Dirichlet	
mRS 0–1	8	0–18		[10]
mRS 2–3	23	0–50		[10]
mRS 4–5	40	30–50		[10]
mRS 6	29	15–45		[10]
mRS after Secondary ICH			Dirichlet	
mRS 0–1	69.4	30–100		[11], [12]
mRS 2–3	22.4	0–50		[11], [12]
mRS 4–5	3.5	0–20		[11], [12]
mRS 6	4.7	0–20		[11], [12]
Vascular anomaly, %	45	0–70		[9]
Primary ICH re-bleed, % per cycle	2.08	0–6	Beta	[46]
Secondary ICH re-bleed, % per cycle	3.92	0–10	Beta	[13]
Relative Risk of re-bleed after repair of vascular anomaly	0.35	0–2	Log	[13], [14]
**Costs (2011 USD)**				
CTA	425	100–1500	Normal	[47]
DSA	1100	450–2000	Normal	[48]
Hospitalization, Secondary ICH	85,400	10,000–300,000	LogNormal	[20]
Hospitalization, Primary ICH & missed lesion	25,300	3,000–120,000	LogNormal	[20]
			LogNormal	
Long-term care, mRS 2–3	8,438	0–20,000	LogNormal	[15]–[19], [49]
Long-term care, mRS 4–5, 1^st^ year	71,428	30,000–150,000	LogNormal	[15]–[19], [49]
Long-term care, mRS 4–5, 2^nd^ year	37,140	10,000–90,000	LogNormal	[15]–[19], [49]
**Utility values**				
mRS 0–1	0.9	0.2–1.0	Beta	[21]
mRS 2–3	0.75	0–1.0	Beta	[21]
mRS 4–5	0.25	−0.1–1.0	Beta	[21]
mRS 6	0			
Discount Rate (%)	3	0–8	Not included	[22]

The range presented is for the one-way sensitivity analysis. The relative risk is for re-bleed after the lesion is fixed. CTA = CT Angiography; DSA = digital subtraction angiogram; ICH = intracerebral hemorrhage; mRS  =  modified Rankin Scale score; NCCT = non-contract head CT; PSA = probabilistic sensitivity analysis; RR = relative risk; USD = United States Dollars.

The hypothetical cohort presented with ICH on the NCCT. The etiology could be either primary (e.g. hypertensive) or secondary ICH relating to an underlying vascular lesion. Hemorrhage as a result of tumor was not included for model simplicity. Any lesion identified by DSA or CTA was considered successfully treated. If a recurrent primary or secondary hemorrhage occurred, we assumed that all subjects underwent DSA and any previously missed lesions would then be successfully treated. Adverse outcomes following DSA in which there was no successful diagnostic treatment were limited to permanent disabling neurological deficits for model simplicity. We assumed that permanent disabling deficits due to the DSA were not separated from permanent disabling deficits due to treatment and were thus captured sufficiently in the mRS distribution following treatment.

### Probabilities

There is a paucity of large randomized trial data for outcomes estimates, thus a combination of sources were used (Table 1). The probability of a vascular anomaly was taken from prior studies and varied with age.[9] Bayes revision was then used with the sensitivity and specificity of NCCT (and/or CTA as appropriate) to determine the post-test probability of a lesion. The distribution of modified Rankin Scale scores after primary ICH was modeled according to the FAST trial.[10] Sample weighted outcomes after secondary ICH were used.[11], [12]

We assumed one rate of recurrent secondary ICH and used a relative risk term to change the likelihood of recurrent hemorrhage with treatment. The relative risk was calculated as the incidence of hemorrhage after treatment based on a meta-analysis divided by the incidence of hemorrhage for the natural history of known vascular lesions.[13], [14]

### Costs and Health Care Utilization

We used a payer perspective. Direct medical costs included the costs of hospitalization, rehabilitation or long-term care, CTA and DSA.[15]–[19] Charges for the hospitalization were converted to costs using data and cost-to-charge ratios from 2009 Nationwide Inpatient Sample (NIS), Healthcare Cost and Utilization Project (HCUP), Agency for Healthcare Research and Quality.[20] The cost of a hospitalization was based on whether or not a lesion was repaired. Repair was identified using ICD-9 procedure codes: 39.72, 39.51, 39.53 with ICD-9 diagnosis code 431. All prices were adjusted to 2011 USD using the Consumer's Price Index for medical care.

A positive CTA implies a readily visible, unequivocal abnormality with lesions proceeding to neurosurgical or endovascular therapy as arbitrated by the physician. We assumed that if endovascular therapy is decided upon, selective DSA of the vessel(s) of interest is undertaken limiting exposure to other vessels. Therefore a separate DSA for diagnosis of the CTA-confirmed abnormality is not performed prior to the planned therapy. Similarly, if a surgical treatment is considered appropriate following diagnostic CTA we assume the patient goes to the operating room without a pre-procedural diagnostic DSA.

If CTA correctly identified a vascular anomaly, the subject underwent successfully treatment without a confirmatory DSA, reflecting widespread clinical practice. The cost structure was:

CTA identified vascular anomaly (true positive):
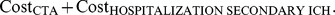



False negative CTA results were assumed to have the cost structure of primary ICH with their treatment plan based on the primary ICH assumption. This cost structures was:

CTA does not detect a vascular anomaly (false negative):
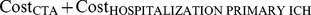



The remaining cost structures were:

CTA identified vascular anomaly (false positive):
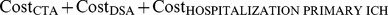



CTA does not detect a vascular anomaly (true negative):
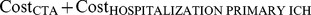



For the cohort in branch (d): high or indeterminate suspicion of a vascular anomaly based on NCCT, we assumed that the DSA cohort incurred the cost of DSA in addition to their hospitalization cost.

### Utility

Utility is the desirability or preference for one health state over another. Perfect health has a utility of one while death is zero. Negative values are allowed representing a state worse than death. Quality adjusted life years (QALYs) are the utility of a health state multiplied by the duration of the health state summed over a lifetime. Utility values were adapted from the *Factor Seven for Acute Hemorrhagic Stroke* trial which measured quality of life at 90 days.[21]

### Analysis

Future costs and outcomes were discounted at 3%.[22] The incremental cost-effectiveness ratio (ICER) is the average net costs of branches a, b or c divided by the net QALYs from branch d of a Markov cohort. For example [($_CTA ALL_ - $_DSA high/indeterminate_) per (QALY_CTA ALL_ – QALY_DSA high/indeterminate_)]. Assumptions about each individual model variable were tested by a one-way sensitivity analysis. A probabilistic sensitivity analysis was used to estimate second order uncertainty about cost, probability and utility data (n = 1000 samples).

## Results

### Case Vignettes

**Figure 2:**
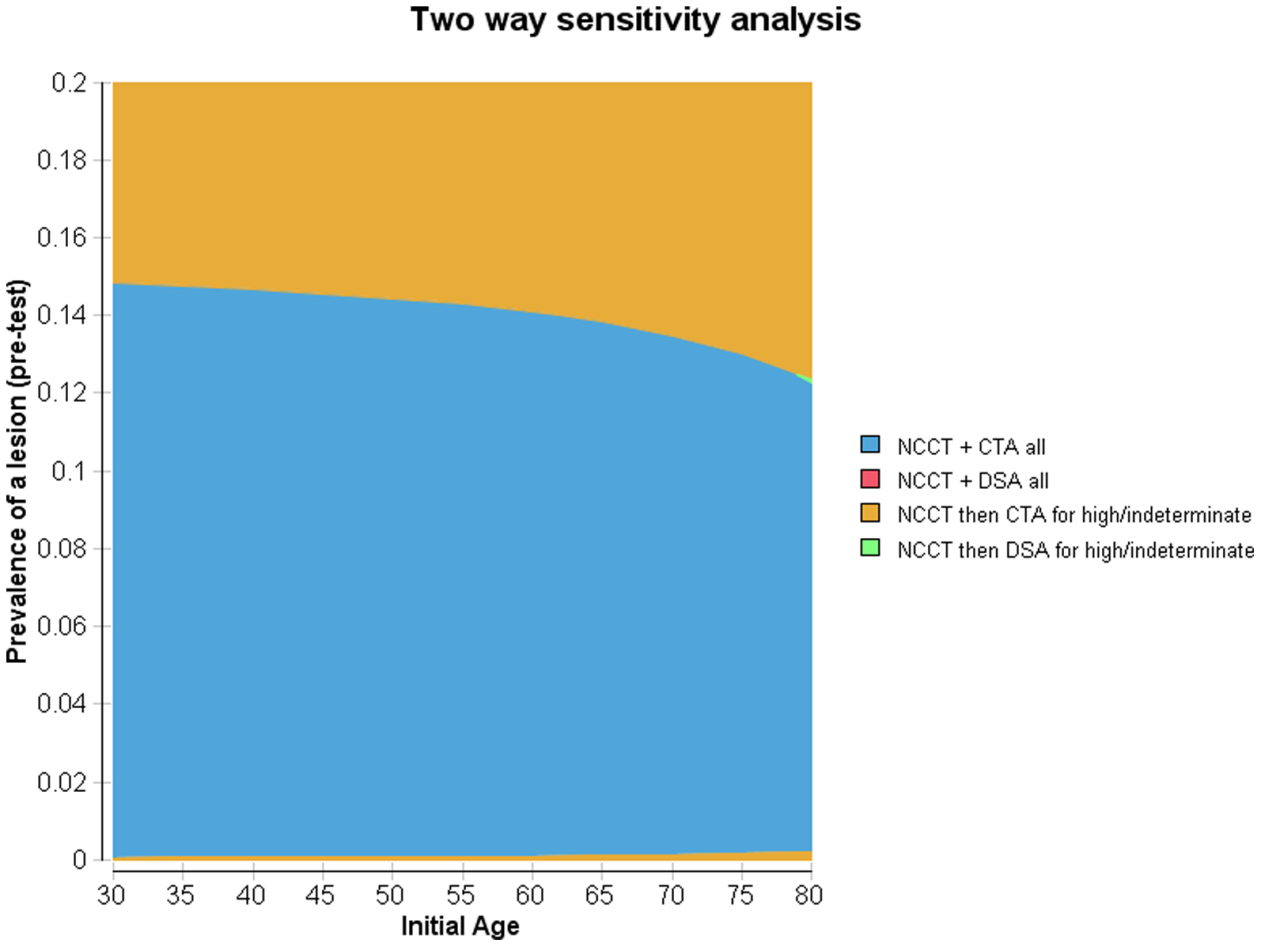
Two-way sensitivity analysis. The prevalence of a lesion and cohort age were tested in a two-way deterministic sensitivity analysis with a willingness-to-pay of $100,000/QALY. The optimal imaging strategy - CTA of the entire cohort (blue), CTA for high or indeterminate suspicion of a lesion on NCCT (orange) and DSA for a high or indeterminate suspicion of a lesion on NCCT (green) – depends on the age and probability of a lesion.

Since the probability of identifying a vascular lesion varies with age, we conducted a two-way sensitivity analysis varying these inputs to discern the optimal imaging strategy for different combinations of these two inputs (Figure 2).[9] Three case vignettes are presented to demonstrate which imaging pathway is the optimal choice. Optimal choice balanced the lifetime costs of care with lifetime outcomes for a willingness-to-pay of $100,000/QALY.

(1) *A 40-year old presented with ICH on NCCT and a 20% probability of an underlying lesion.*

Using 40 years on the x-axis and 20% on the y-axis of Figure 2, the optimal pathway was NCCT with CTA for a high or indeterminate suspicion of a lesion.

(2) *A 25-year old presented with ICH on NCCT and a 40% probability of an underlying lesion*

With a high index of suspicion for a lesion, the optimal pathway was NCCT with CTA for a high or indeterminate suspicion of a lesion.

(3) *A 60-year old presented with ICH on NCCT and a 2% probability of an underlying lesion.*

With a low index of suspicion for a lesion, the optimal pathway was NCCT with CTA for all persons.

Broadly speaking, when the probability of an underlying lesion is ≥15%, stratified CTA for high or indeterminate NCCT is the optimal strategy regardless of age using net monetary benefits with a willingness-to-pay of $100,000 per QALY. When probability of an underlying lesion was below 15% the dominant strategy was NCCT and CTA in all patients below 60 years of age. Above 60 years, stratified CTA for high or indeterminate NCCT became cost effective for a lesion probability of 13–14%.

Several factors contribute to the value of the ICER beyond the costs of the initial screening strategies accounting for cost-effectiveness of CTA screening in all older patients (scenario 3) but selectively applying CTA in the younger cohort. ICERs and net benefit models used in these analyses reflect the contribution of many factors including total lifetime costs, health related outcomes, the balance of the proportion of primary and secondary ICH as well as the age of the cohort. The numerator of an ICER is the incremental net costs of a strategy accumulated over a lifetime following hospitalization, long-term care and adverse events, thus the numerator reflects more than just the incremental cost of DSA or CTA. Compared to secondary hemorrhage primary hemorrhage has, relatively speaking, (a) a worse distribution of mRS scores for outcomes, (b) lower hospitalization costs but higher long-term care costs experienced due to greater disability, (c) and lower overall utility. There is also additional burden (higher probability of re-bleed if a lesion is not fixed, cost and outcomes) for missed lesions on the CTA. The individual influence of each of these factors is explored by using sensitivity analyses.

### One-way Sensitivity Analyses

**Figure 3:**
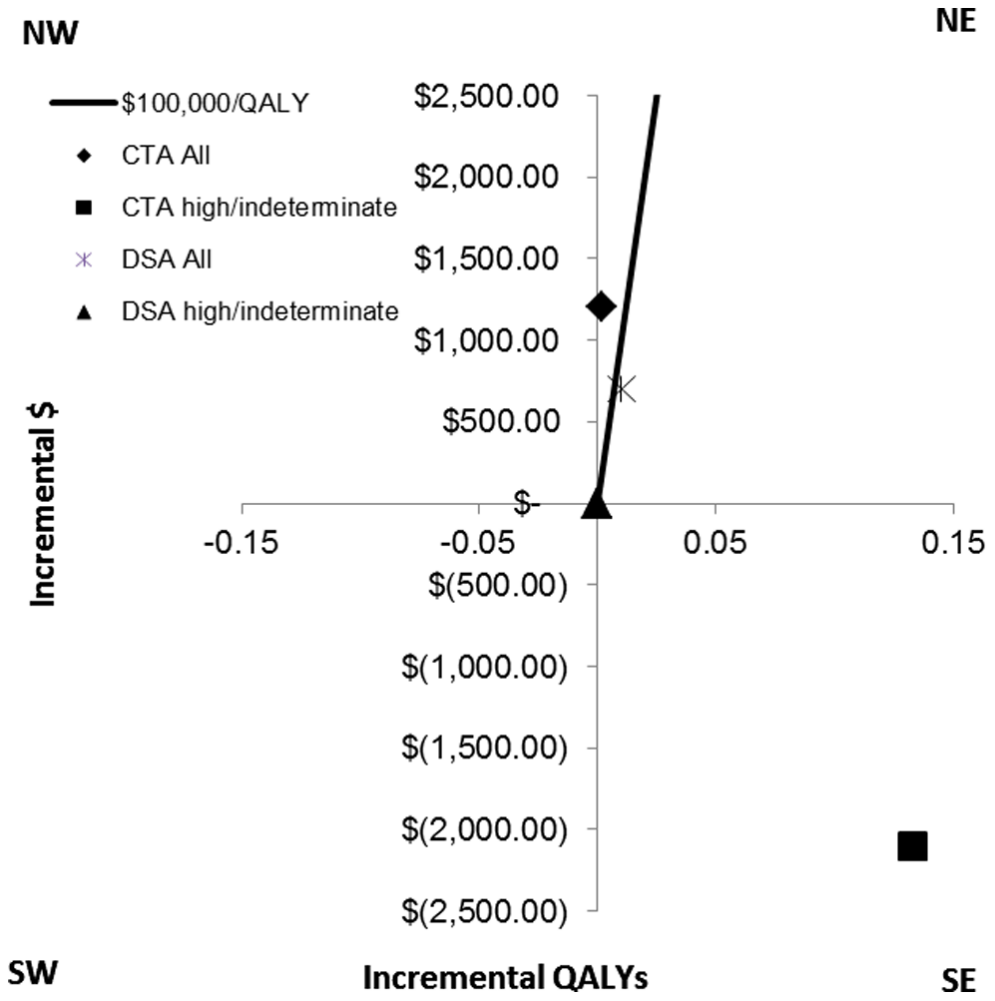
Base Case graphs. The base case analyses are presented on a two-dimensional graph showing the net costs and net QALYs for CTA of the entire cohort (diamond), CTA for high or indeterminate suspicion of a lesion on NCCT (square), DSA for the entire cohort (asterisk) and DSA for those with a high or indeterminate suspicion of a lesion on NCCT (triangle).

One-way sensitivity analyses were conducted to identify which individual model inputs influenced the ICER. To complete these analyses, we specified a base case analysis with a 40-year old cohort the prevalence of vascular anomaly reported for this age.[9] The strategy of using stratified CTA for a 40-year old cohort with a high or indeterminate NCCT had lower net costs (-$2,100) and greater QALYs (0.133) than stratified DSA for those with a high or indeterminate NCCT making it the dominant screening strategy (Figure 3 and Table S1 in File S1). DSA screening of the entire cohort had an ICER of $70,000 per QALY while the ICER for CTA screening of the entire cohort was $600,000 per QALY.

To address the uncertainty around the likelihood of finding a vascular anomaly, we tested our model by varying this probability in a 40-year old cohort. The choice of optimal imaging strategy was sensitive to the probability of a vascular lesion (Table 2). When the probability of a lesion was ≤6%, CTA screening of the entire 40-year old cohort became the dominant option with the lowest costs and greatest QALYs (dark green) compared to all other imaging strategies. CTA screening of the entire cohort was a reasonable option, for up to a 15% probability of a lesion (light green). Stratified CTA following high or indeterminate NCCT was the optimal strategy compared to all others tested when the probability of an underlying lesion was 16–60%.

**Table 2:** One way sensitivity analysis for the probability of a lesion in a 40-year old cohort.

Probability of a Lesion	NCCT + CTA All	NCCT + CTA high/indeterminate	NCCT + DSA All	NCCT + DSA high/indeterminate
1–15%	**Dominant**	*Not optimal*	*Not optimal*	Comparator
16–17%	**Dominant**	**Dominant**	*Not optimal*	Comparator
18–20%	<$100,000/QALY	**Dominant**	*Not optimal*	Comparator
21–33%	*Not optimal*	**Dominant**	*Not optimal*	Comparator
34–60%	*Not optimal*	**Dominant**	<$100,000/QALY	Comparator
vs all other strategies				
1–6%	**Dominant**			
16–60%		**Dominant**		

Bold: The strategy is dominant (less costly and more QALYs than the comparator). Normal: The ICER falls in an acceptable range. Italics: Not optimal - the strategy is dominated, in the southwest quadrant, or in the northwest quadrant above the $100,000 per QALY line.

Changing the initial age of the cohort also influenced the optimal imaging strategy for a 45% probability of a lesion. Stratified CTA following high or indeterminate NCCT was the dominant strategy compared to all other strategies tested for ages 10–40 and 51–80 (Tables S2 in File S1 and S3 in File S1). DSA screening of the entire cohort became cost-ineffective (ICER >$100,000 per QALY) at age ≥41 years; CTA screening of the entire cohort became cost-effective (ICER <$100,000 per QALY) only for ages 41–50. However, this change is likely due to the 8% probability of a lesion, rather than age itself (see Two-way Sensitivity Analysis).[9]

Changes in the assumptions of two model inputs increased ICER of DSA screening of the entire cohort beyond $100,000 per QALY. DSA screening of the entire cohort became cost-ineffective if either the re-bleed for secondary ICH was <3% per cycle or the relative risk of re-bleed after treatment of the vascular anomaly was >0.42. The results of other one-way sensitivity analyses were presented in Table S2 in File S1.

The relative risk of a re-bleed was tested with the re-bleed rate as another two-way sensitivity analysis. Stratified CTA for high or indeterminate suspicion of lesion following NCCT was the optimal strategy across all combinations using a net monetary benefit model with a willingness-to-pay of either $50,000 or $100,000 per QALY (data not shown).

### Probabilistic Sensitivity Analysis

**Figure 4:**
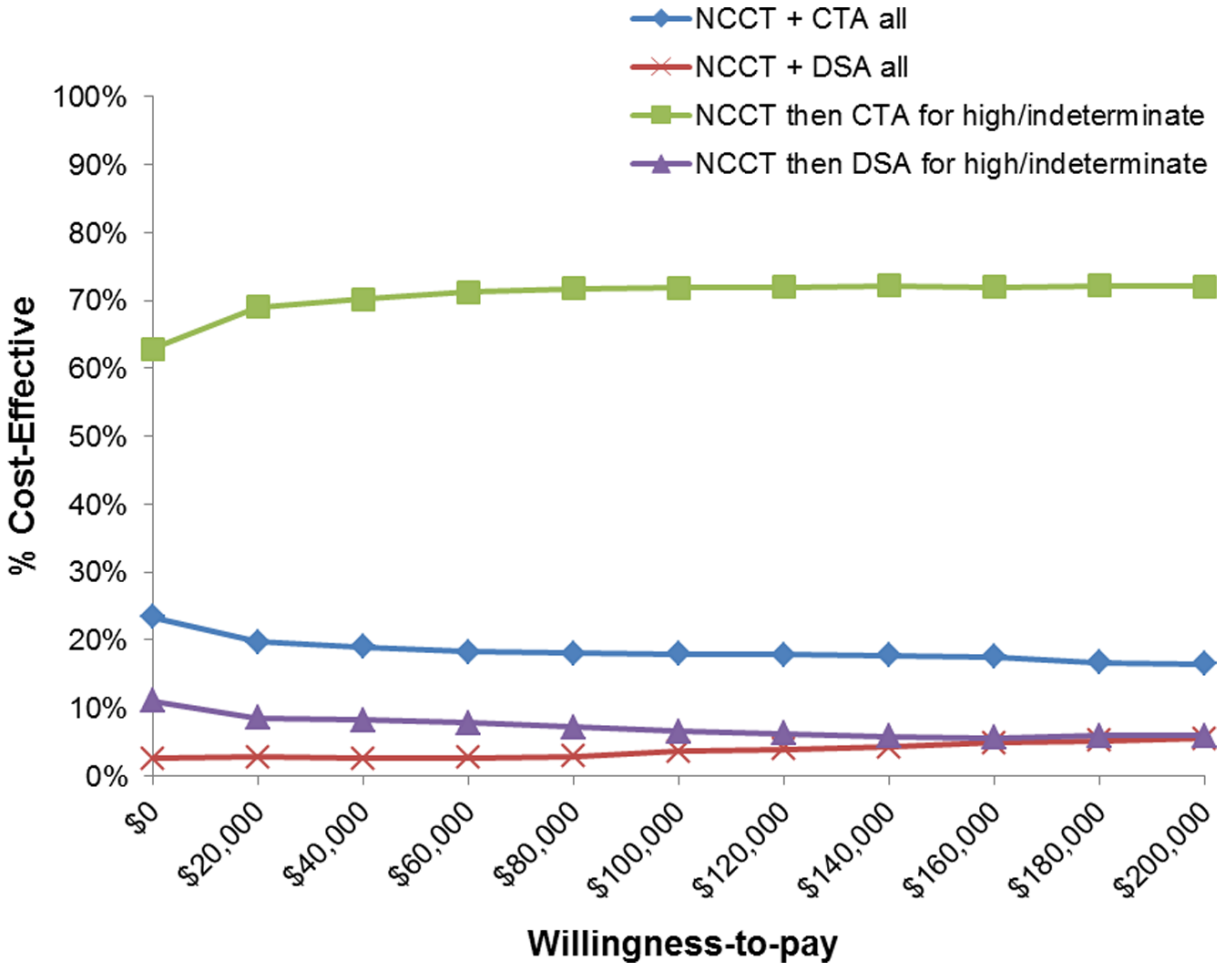
Probabilistic sensitivity analysis. Variables in the model were sampled simultaneously to show the probability that a given strategy was optimal strategy in relation to the willingness-to-pay (x-axis) using net monetary benefits calculations.

Monte Carlo simulations showed that stratified CTA for high or indeterminate suspicion of a lesion on NCCT was the optimal screening strategy 70–72% of the time varying slightly with the willingness-to-pay (Figure 4).

## Discussion

The most cost effective strategy for screening of ICH patients is dependent on both age and underlying vascular lesion prevalence. However, a CTA strategy for all patients was the optimal imaging strategy regardless of age when the risk of an underlying vascular lesion was below 12%. This strategy remained optimal in younger patients (<∼35 years) with a risk below 15%. When the risk exceeds 15%, a strategy first stratifying patients into high and indeterminate risk categories by NCCT prior to CTA is the preferred strategy regardless of age. Furthermore, the results of the probabilistic sensitivity analysis which showed stratified CTA is an optimal screening strategy in ∼70% of the simulations.

In a large recent retrospective study 623 patients were divided according to their NCCT scan appearances into low, indeterminate and high risk for underlying vascular lesions. High risk scans constituted only a minority of ICH presentations (19/623; 3%). The large majority of patients demonstrated indeterminate (421; 68%) scans, while low risk scans constituted the remaining 29%. More recent studies confirm frequencies of high, intermediate and low risk scans of 2–11%, 42–61% and 32–36% respectively.[23]–[25] Acknowledging the need to further investigate patients with indeterminate risk and confirm NCCT suspicion in the high risk group, up to 90% of the ICH population would still require further angiographic imaging with this stratification approach. NCCT stratification would spare only the 11–30% defined as low risk from angiographic imaging but yield a 1.6–8%% false negative rate.[2], [24], [26] An external validation of attempts to combine NCCT risk stratification with clinical variables including age, gender, hypertensive/anticoagulation status to define ‘low risk’ [1], [2], [7], [9], [24], [26] yielded an AUCs of 0.82 for predicting underlying vascular lesion presence with NCCT stratification faring as well as a combined imaging/radiological score.[27] The average performance of NCCT therefore provides a compelling argument for consideration of a CTA base screening algorithm given its sensitivity and specificity ranging from 92–100% for secondary vascular lesion detection.[2]–[5], [7]

The notion of negligible risk in ‘typical hypertensive’ locations is largely derived from a prospective study of 206 patients examined by DSA reporting a 0% prevalence of secondary vascular lesions for basal ganglia ICH in older (>45 yrs) hypertensive patients.[9] A reappraisal of the study, however suggests that this group remains worthy of further angiographic study as the caudate head was erroneously omitted from the study's basal ganglia definition. The authors actually demonstrated 1 caudate hemorrhage secondary to an aneurysm in an old hypertensive patient. Rightful inclusion of the caudate head into the basal ganglia definition results in a secondary vascular lesion in 1/29 or 2.4% of older patients with ‘typical hypertensive’ locations. Similarly, older hypertensive patients with any ICH location demonstrated an angiographic yield of 11% (age >50 yrs) in a prospective and 9.5% (age >46 yrs) in a retrospective study.[2], [8] Finally a recent study from a center adopting a more liberal DSA approach demonstrated a 15% incidence of vascular lesions in a patient subgroup that was hypertensive (or impaired coagulation; 72%), older and more likely to present with a low probability scan (56%). In this study 7% of deep location ICH was associated with a secondary vascular lesion. According to our results the combination of age >40 and low vascular lesion prevalence suggests that this specific patient group would be best served with a combined NCCT and CTA strategy. Indeed a CTA–all-patients strategy was recommended by the same group.[28]

The reliance on DSA as a reference standard to exclude vascular lesions in ICH before the maturation of CTA as an accepted technique undoubtedly resulted in both a selection and verification bias. It is noteworthy that prospective angiographic studies show a higher (34–53%) prevalence of secondary ICH than the 20% commonly cited.[7]–[9], [29] CTA is associated with a lower overall patient risk than DSA and can be performed safely with limited morbidity and mortality. Several studies report the safety of contrast use in the stroke population with the incidence of contrast induced nephropathy (CIN) below 5%.[30]–[36] The American college of radiology states that the concern for the development of CIN is a relative but not absolute contraindication to the administration of intravascular contrast.[37] The role of contrast in the development of nephropathy is overstated with only 1 of 8 studies that included a control group showing a link between contrast and CIN. [30], [32], [38]–[43]

Limitations of this study relate to the paucity of prospective data available on which to base the incidence of secondary vascular lesions. Additionally, new information about outcomes by age, volume and etiology, primary vs secondary, would help strengthen the model. While the included study is the only prospective study available that utilizes the reference standard procedure DSA, the single center nature and non-North American demographic studied could limit its application to other population. Models of cost effectiveness rely on underlying assumptions that, while tested, remain assumptions. However the purpose of any cost-effectiveness model is to help frame questions and inform medical decision making.[44]

In summary a strategy imaging all patients with NCCT and CTA is optimal when the risk of an underlying lesion is below 10% whereas an NCCT stratification strategy is dominant where risk exceeds this threshold.

## Supporting Information

File S1 **Tables S1–S3.** Table S1. Base Case Analysis. The cohort was 40 years and had a 45% probability of a vascular anomaly. Table S2. One way sensitivity analyses (except age and probability of a lesion). *Initial age 41–50 has to do with the probability of an underlying lesion. The look-up table used for the analysis had a lower probability for this decade. Bold: The strategy is dominant (less costly and more QALYs than the comparator). Table S3. One-way sensitivity analysis for age. Normal: The ICER falls in an acceptable range. Italics: Not optimal - the strategy is dominated, in the southwest quadrant, or in the northwest quadrant above the $100,000 per QALY line.
(DOCX)

## References

[1] Morgenstern LB, Hemphill JC III, Anderson C, Becker K, Broderick JP, et al (2010) Guidelines for the management of spontaneous intracerebral hemorrhage: a guideline for healthcare professionals from the American Heart Association/American Stroke Association. Stroke 41: 2108–2129. 20651276 10.1161/STR.0b013e3181ec611b PMC4462131

[2] Delgado Almandoz JE, Schaefer PW, Forero NP, Falla JR, Gonzalez RG, et al (2009) Diagnostic accuracy and yield of multidetector CT angiography in the evaluation of spontaneous intraparenchymal cerebral hemorrhage. AJNR Am J Neuroradiol 30: 1213–1221. 19342546 10.3174/ajnr.A1546 PMC7051335

[3] Yeung R, Ahmad T, Aviv RI, de Tilly LN, Fox AJ, et al (2009) Comparison of CTA to DSA in determining the etiology of spontaneous ICH. Can J Neurol Sci 36: 176–180. 19378710 10.1017/s0317167100006533

[4] Romero JM, Artunduaga M, Forero NP, Delgado J, Sarfaraz K, et al (2009) Accuracy of CT angiography for the diagnosis of vascular abnormalities causing intraparenchymal hemorrhage in young patients. Emerg Radiol 16: 195–201. 19132425 10.1007/s10140-008-0785-3

[5] Yoon DY, Chang SK, Choi CS, Kim WK, Lee JH (2009) Multidetector row CT angiography in spontaneous lobar intracerebral hemorrhage: a prospective comparison with conventional angiography. AJNR Am J Neuroradiol 30: 962–967. 19193746 10.3174/ajnr.A1471 PMC7051684

[6] Khosravani H, Mayer SA, Demchuk AM, Jahromi BS, Gladstone DJ, et al.. (In Press) Emergency Noninvasive Angiography for Acute Intracerebral Hemorrhage. AJNR Am J Neuroratiol. 10.3174/ajnr.A3296 PMC8051448 23124634

[7] Wong GK, Siu DY, Abrigo JM, Poon WS, Tsang FC, et al (2011) Computed tomographic angiography and venography for young or nonhypertensive patients with acute spontaneous intracerebral hemorrhage. Stroke 42: 211–213. 21088241 10.1161/STROKEAHA.110.592337

[8] Halpin SF, Britton JA, Byrne JV, Clifton A, Hart G, et al (1994) Prospective evaluation of cerebral angiography and computed tomography in cerebral haematoma. J Neurol Neurosurg Psychiatry 57: 1180–1186. 7931378 10.1136/jnnp.57.10.1180 PMC485483

[9] Zhu XL, Chan MS, Poon WS (1997) Spontaneous intracranial hemorrhage: which patients need diagnostic cerebral angiography? A prospective study of 206 cases and review of the literature. Stroke 28: 1406–1409. 9227692 10.1161/01.str.28.7.1406

[10] Mayer SA, Brun NC, Begtrup K, Broderick J, Davis S, et al (2005) Recombinant activated factor VII for acute intracerebral hemorrhage. N Engl J Med 352: 777–785. 15728810 10.1056/NEJMoa042991

[11] Hartmann A, Mast H, Mohr JP, Koennecke HC, Osipov A, et al (1998) Morbidity of intracranial hemorrhage in patients with cerebral arteriovenous malformation. Stroke 29: 931–934. 9596237 10.1161/01.str.29.5.931

[12] van BJ, Lovelock CE, Cordonnier C, Rothwell PM, Klijn CJ, et al (2009) Outcome after spontaneous and arteriovenous malformation-related intracerebral haemorrhage: population-based studies. Brain 132: 537–543. 19042932 10.1093/brain/awn318

[13] Ondra SL, Troupp H, George ED, Schwab K (1990) The natural history of symptomatic arteriovenous malformations of the brain: a 24-year follow-up assessment. J Neurosurg 73: 387–391. 2384776 10.3171/jns.1990.73.3.0387

[14] van BJ, van der Worp HB, Buis DR, Al-Shahi SR, Kappelle LJ, et al (2011) Treatment of brain arteriovenous malformations: a systematic review and meta-analysis. JAMA 306: 2011–2019. 22068993 10.1001/jama.2011.1632

[15] Oster G, Huse DM, Lacey MJ, Epstein AM (1994) Cost-effectiveness of ticlopidine in preventing stroke in high-risk patients. Stroke 25: 1149–1156. 8202972 10.1161/01.str.25.6.1149

[16] Kuntz KM, Kent KC (1996) Is carotid endarterectomy cost-effective? An analysis of symptomatic and asymptomatic patients. Circulation 94: II194–II198. 8901745

[17] Cronenwett JL, Birkmeyer JD, Nackman GB, Fillinger MF, Bech FR, et al (1997) Cost-effectiveness of carotid endarterectomy in asymptomatic patients. J Vasc Surg 25: 298–309 discussion 310–291. 9052564 10.1016/s0741-5214(97)70351-3

[18] Yin D, Carpenter JP (1998) Cost-effectiveness of screening for asymptomatic carotid stenosis. J Vasc Surg 27: 245–255. 9510279 10.1016/s0741-5214(98)70355-6

[19] Post PN, Kievit J, van Baalen JM, van den Hout WB, van Bockel JH (2002) Routine duplex surveillance does not improve the outcome after carotid endarterectomy: a decision and cost utility analysis. Stroke 33: 749–755. 11872899 10.1161/hs0302.103624

[20] HCUP Nationwide Inpatient Sample (NIS). Healthcare cost and Utilization Project (HCUP). 2009.Agency for Healthcare Research and Quality, Rockville, MD.

[21] Christensen MC, Mayer S, Ferran JM (2009) Quality of life after intracerebral hemorrhage: results of the Factor Seven for Acute Hemorrhagic Stroke (FAST) trial. Stroke 40: 1677–1682. 19265046 10.1161/STROKEAHA.108.538967

[22] (1996) Cost-effectiveness in health and medicine. Gold MB, Siegel JE, Rusell LB, Weinstein MC, eds. New York: Oxford University Press.

[23] Kadkhodayan Y, Cross DT III, Derdeyn CP, Moran CJ (2007) Carotid angioplasty and stenting in the elderly. Neuroradiology 49: 933–938. 17657482 10.1007/s00234-007-0278-1

[24] Delgado Almandoz JE, Jagadeesan BD, Moran CJ, Cross DT III, Zipfel GJ, et al (2012) Independent validation of the secondary intracerebral hemorrhage score with catheter angiography and findings of emergent hematoma evacuation. Neurosurgery 70: 131–140 discussion 140. 21808219 10.1227/NEU.0b013e31822fbf43

[25] van Asch CJ, Velthuis BK, Greving JP, van Laar PJ, Rinkel GJ, et al (2013) External validation of the secondary intracerebral hemorrhage score in The Netherlands. Stroke 44: 2904–2906. 23920015 10.1161/STROKEAHA.113.002386

[26] Kadkhodayan Y, Delgado Almandoz JE, Kelly JE, Kale SP, Jagadeesan BD, et al (2012) Yield of catheter angiography in patients with intracerebral hemorrhage with and without intraventricular extension. J Neurointerv Surg 4: 358–363. 21990524 10.1136/neurintsurg-2011-010077

[27] Gerlach UA, Pascher A (2012) Technical advances for abdominal wall closure after intestinal and multivisceral transplantation. Current Opinion in Organ Transplantation 17: 258–267. 22476222 10.1097/MOT.0b013e3283534d7b

[28] Kadkhodayan Y, Delgado Almandoz JE, Kelly JE, Kale SP, Jagadeesan BD, et al (2012) Yield of catheter angiography in patients with intracerebral hemorrhage with and without intraventricular extension. J Neurointervent Surg 4: 358–363. 10.1136/neurintsurg-2011-010077 21990524

[29] Toffol GJ, Biller J, Adams HP Jr, Smoker WR (1986) The predicted value of arteriography in nontraumatic intracerebral hemorrhage. Stroke 17: 881–883. 3764958 10.1161/01.str.17.5.881

[30] Oleinik A, Romero JM, Schwab K, Lev MH, Jhawar N, et al (2009) CT angiography for intracerebral hemorrhage does not increase risk of acute nephropathy. Stroke 40: 2393–2397. 19461032 10.1161/STROKEAHA.108.546127 PMC2726774

[31] Tippins RB, Torres WE, Baumgartner BR, Baumgarten DA (2000) Are screening serum creatinine levels necessary prior to outpatient CT examinations? Radiology 216: 481–484. 10924574 10.1148/radiology.216.2.r00au23481

[32] Lima FO, Lev MH, Levy RA, Silva GS, Ebril M, et al (2010) Functional contrast-enhanced CT for evaluation of acute ischemic stroke does not increase the risk of contrast-induced nephropathy. AJNR Am J Neuroradiol 31: 817–821. 20044502 10.3174/ajnr.A1927 PMC2873111

[33] Olsen JC, Salomon B (1996) Utility of the creatinine prior to intravenous contrast studies in the emergency department. J Emerg Med 14: 543–546. 8933312 10.1016/s0736-4679(96)00125-4

[34] Aulicky P, Mikulik R, Goldemund D, Reif M, Dufek M, et al (2010) Safety of performing CT angiography in stroke patients treated with intravenous thrombolysis. J Neurol Neurosurg Psychiatry 81: 783–787. 19965846 10.1136/jnnp.2009.184002

[35] Hopyan JJ, Gladstone DJ, Mallia G, Schiff J, Fox AJ, et al (2008) Renal safety of CT angiography and perfusion imaging in the emergency evaluation of acute stroke. AJNR Am J Neuroradiol 29: 1826–1830. 18719035 10.3174/ajnr.A1257 PMC8118945

[36] Krol AL, Dzialowski I, Roy J, Puetz V, Subramaniam S, et al (2007) Incidence of radiocontrast nephropathy in patients undergoing acute stroke computed tomography angiography. Stroke 38: 2364–2366. 17600231 10.1161/STROKEAHA.107.482778

[37] ACR Committee on Drugs and Contrast Medoa (2013) ACR Manual on Contrast Media. Version 9. American College of Radiology.

[38] Bruce RJ, Djamali A, Shinki K, Michel SJ, Fine JP, et al (2009) Background fluctuation of kidney function versus contrast-induced nephrotoxicity. AJR Am J Roentgenol 192: 711–718. 19234268 10.2214/AJR.08.1413

[39] Cramer BC, Parfrey PS, Hutchinson TA, Baran D, Melanson DM, et al (1985) Renal function following infusion of radiologic contrast material. A prospective controlled study. Arch Intern Med 145: 87–89. 3882071

[40] Heller CA, Knapp J, Halliday J, O'Connell D, Heller RF (1991) Failure to demonstrate contrast nephrotoxicity. Med J Aust 155: 329–332. 1895978 10.5694/j.1326-5377.1991.tb142293.x

[41] Langner S, Stumpe S, Kirsch M, Petrik M, Hosten N (2008) No increased risk for contrast-induced nephropathy after multiple CT perfusion studies of the brain with a nonionic, dimeric, iso-osmolal contrast medium. AJNR Am J Neuroradiol 29: 1525–1529. 18524974 10.3174/ajnr.A1164 PMC8119048

[42] McGillicuddy EA, Schuster KM, Kaplan LJ, Maung AA, Lui FY, et al (2010) Contrast-induced nephropathy in elderly trauma patients. J Trauma 68: 294–297. 20154540 10.1097/TA.0b013e3181cf7e40

[43] Tremblay LN, Tien H, Hamilton P, Brenneman FD, Rizoli SB, et al (2005) Risk and benefit of intravenous contrast in trauma patients with an elevated serum creatinine. J Trauma 59: 1162–1166 discussion 1166–1167. 16385295 10.1097/01.ta.0000194694.71607.0c

[44] Young KC, Kelly AG, Holloway RG (In Press) Reading a Cost-effectiveness or Decision Analysis Study: 5 Things to Consider. Neurology: Clinical Practice 3: 413–420. 24175157 10.1212/CPJ.0b013e3182a78fd8 PMC3806934

[45] Delgado Almandoz JE, Schaefer PW, Goldstein JN, Rosand J, Lev MH, et al (2010) Practical scoring system for the identification of patients with intracerebral hemorrhage at highest risk of harboring an underlying vascular etiology: the Secondary Intracerebral Hemorrhage Score. AJNR AmJNeuroradiol 31: 1653–1660. 10.3174/ajnr.A2156 PMC3682824 20581068

[46] Vermeer SE, Algra A, Franke CL, Koudstaal PJ, Rinkel GJ (2002) Long-term prognosis after recovery from primary intracerebral hemorrhage. Neurology 59: 205–209. 12136058 10.1212/wnl.59.2.205

[47] (2012) Reimbursement Information for CT Perfusion in the Diagnosis of Acute Stroke. Available: http://www3.gehealthcare.com/en/Products/~/media/Downloads/us/Product/Reimbursement/Customer-Advisories/GEHealthcare-Customer-Advisory_CT-Perfusion-Diagnosis-Acute-Stroke-Reimbursement-Info-2012.pdf. Accessed March 21, 2013.

[48] (2011) 2011 Medicare Physician Fee Schedule Payment Rates: Angiography/Vascular Procedures. Available: http://www.medical.siemens.com/siemens/en_US/rg_marcom_FBAs/files/Reimbursement/Angio_2011_Final_MPFS_Payment_Rates.pdf. Accessed March 21, 2013.

[49] Kilaru S, Korn P, Kasirajan K, Lee TY, Beavers FP, et al (2003) Is carotid angioplasty and stenting more cost effective than carotid endarterectomy? J Vasc Surg 37: 331–339. 12563203 10.1067/mva.2003.124

