# Supplementary material for: The Cost-Utility of CT Angiography and Conventional Angiography for People Presenting with Intracerebral Hemorrhage
Source: PLoS One. 2014 May 13;9(5):e96496. doi: 10.1371/journal.pone.0096496 (PMC4019473; doi:10.1371/journal.pone.0096496)
Supplement: File S1 — Tables S1–S3. Table S1. Base Case Analysis. The cohort was 40 years and had a 45% probability of a vascular anomaly. Table S2. One way sensitivity analyses (except age and probability of a lesion). *Initial age 41–50 has to do with the probability of an underlying lesion. The look-up table used for the analysis had a lower probability for this decade. Bold: The strategy is dominant (less costly and more QALYs than the comparator). Table S3. One-way sensitivity analysis for age. Normal: The ICER falls in an acceptable range. Italics: Not optimal - the strategy is dominated, in the southwest quadrant, or in the northwest quadrant above the $100,000 per QALY line. (DOCX) [file pone.0096496.s001.docx]

**Table S1**.

|  | **Cost** | **Incremental Cost** | **QALYs** | **Incremental QALYs** | **Marginal $ per QALY (ICER)** |
| --- | --- | --- | --- | --- | --- |
| Intervention |  |  |  |  |  |
| CTA all | $311,900 | $1,200 | 11.635 | 0.002 | $600,000 |
| CTA high/indeterminate NCCT | $308,600 | -$2,100 | 11.766 | 0.133 | Dominant |
| DSA All | $311,400 | $700 | 11.643 | 0.01 | $70,000 |
| Comparator |  |  |  |  |  |
| DSA high/indeterminate NCCT | $310,700 |  | 11.633 |  |  |

**Table S2**.

| **Variable** |  |
| --- | --- |
| **CTA high/indeterminate** | **NE crossing to > $100,000 / QALY** |
| Sensitivity of CTA | ≥ 0.99 |
| Specificity of CTA | ≤ 0.955 |
|  |  |
| **DSA All** | **NE crossing to > $100,000 / QALY** |
| Initial Age | ≥ 41 years |
| Hospitalization, Secondary ICH | ≥ $105,000 |
| Hospitalization, Primary ICH & missed lesion | ≤ $13,000 |
| Long-term care, minor stroke | ≤ $14,000 |
| Utility, mRS 0-1 | ≤ 0.80 |
| Utility, mRS 2-3 | ≥ 0.89 |
| Discount | ≥ 4% |
| Specificity of NCCT | ≥ 0.59 |
| mRS after secondary ICH  0-1, % | > 98% |
| 4-5, % | < 1% |
| 6, % | < 2% |
| Secondary ICH re-bleed, % per cycle | < 3% |
| Relative Risk of re-bleed after repair of vascular anomaly | > 0.42 |
|  |  |
| **CTA All** | **NE crossing to < $100,000 / QALY** |
| Initial Age | 41-50* |
| Hospitalization, Secondary ICH | ≤ $44,300 |
| Sensitivity of NCCT | ≤ 0.87 |
| mRS after secondary ICH  6, % | ≤ 2% |
|  |  |

**Table S3**.

| Initial Age | NCCT + CTA All | NCCT + CTA high/indeterminate | NCCT + DSA All | NCCT + DSA high/indeterminate |
| --- | --- | --- | --- | --- |
| 10-40 | *>$100,000 / QALY* | **Dominant** | <$100,000 / QALY | Comparator |
| 41-50 | **Dominant** | *SW* | *>$100,000 / QALY* | Comparator |
| 51-80 | *>$100,000 / QALY* | **Dominant** | *>$100,000 / QALY* | Comparator |
|  |  |  |  |  |
| vs all other strategies |  |  |  |  |
| 10-40 |  | **Dominant** |  |  |
| 51-80 |  | **Dominant** |  |  |
